# Calculation Derivation and Test Verification of Indirect Tensile Strength of Asphalt Pavement Interlayers at Low Temperatures

**DOI:** 10.3390/ma14206041

**Published:** 2021-10-13

**Authors:** Qian Zhang, Zhihe Fang, Yiheng Xu, Zhao Ma

**Affiliations:** 1 School of Civil Engineering, Xi’an University of Architecture and Technology, Xi’an 710055, China; afzhfzh@163.com (Z.F.); Xuyi_hengd@163.com (Y.X.); Zhao_Ma1@163.com (Z.M.); 2 Shaanxi Key Laboratory of Geotechnical and Underground Space Engineering, Xi’an 710055, China; 3 China Construction Science & Technology Chengdu Co., Ltd., Chengdu 610213, China; 4 Zhejiang Communications Construction Group Co., Ltd., Hangzhou 310051, China

**Keywords:** road engineering, asphalt pavement, indirect tensile strength test, interlayer bond strength, calculation derivation

## Abstract

When the direct tensile test is adopted to determine the interlayer tensile strength of the asphalt pavements, specimen separation or internal cracking often occurs at the bonding area of the loading head, rather than at the interlaminar bonding interface. In view of the tedious and discrete data of the direct tensile test, this paper attempts to introduce an indirect tensile test to determine the interlayer bond strength of asphalt pavement to solve this problem. However, the indirect tensile test method of a binder lacks the corresponding mechanical theory. This paper deduces the calculation formula of the indirect tensile strength of a binder based on elastic theory. A mechanical model of the test was established with the finite element method. In accordance with the two-dimensional elastic theory and the Flamant solution, an analytical solution of tensile stress in the indirect tensile test is proposed through the stress superposition. On this basis, the calculation formula for the indirect tensile strength of the interlaminar bonding is derived according to Tresca’s law. A low-temperature indirect tensile test was designed and conducted to verify the correctness of the formula. By comparing the results of the indirect tensile test and direct tensile test, it is found that the interlaminar strength of the mixture measured by them is similar, and the dispersion of indirect tensile test results is small. The results show that the indirect tensile test can replace the direct tensile test to evaluate the interlaminar tensile strength.

## 1. Introduction

The direct tensile test is often applied to measure the interlayer bonding strength of asphalt pavements [1,2,3]. However, this test method is time-consuming because the specimen needs to be adhered to the loading head using high-quality glue [4]. Even though, the detachment frequently occurs at the glued interface during the test [5]. Besides, a tensile failure may occur in the specimen itself instead of along the interlayer bonding surface.

Considering the defect of the direct tensile test, Ehsan adopted an indirect interlayer tensile test to evaluate the mechanical properties of the cold joints of the asphalt pavement by referring to the direct tensile test and a four-point bending test [6]. The results show that the indirect tensile test is practicable. However, Ehsan did not make a corresponding mechanical theoretical derivation for the indirect tensile test method, only directly following the mechanical theory of the Brazilian disc test proposed by Hondros in 1959 for the test of low tensile strength materials [7]. Therefore, the calculation results are not so resonable. The Brazilian disc is the most widely used approach to test the tensile strength $\sigma_{t}$ of rocks [8,9,10], and was first used by Guo et al. in testing the open fracture toughness of rocks [11]. The difference between this approach and other fracture tests is that it is unnecessary to introduce the crack or the pre-opened groove in the test piece [12]. Nevertheless, due to the high compressive stress generated between the indenter and the specimen, the rock may yield and fracture near the loading point, which is inconsistent with the test principle [13]. That’s why the method yet needs to be improved. Wang et al. solved the problem by improving the Brazilian disk [14,15,16]. Two cuts were introduced on the upper and lower ends of the disk specimen to make two parallel platforms for ease of loading.

By analyzing the loading mode of the disc, Guo et al. found that the concentrated loading mode and the loading plate approach are susceptible to a shear failure at the loading point, while the platform loading mode can better ensure a center cracking of the disc [17]. This is the theoretical premise of the indirect tensile strength test. According to Saint-Venant’s principle in elastic mechanics, if the surface force applied on a portion of the boundary of an object is transformed into a static equivalent surface force with a different distribution (the principal vector is the same, so is the principal moment for the same point), the vicinity stress distribution on the boundary of the object will change significantly, with the effect on the distance being negligible [18]. This implicates that as long as the loading angle of the platform is within a certain range, the above conclusion is believable. The researches are done by Qi [19], Wang [20,21], Khavari P [22], et al. shows that the optimal loading angle is between 20° and 30°. Through the finite element analysis, Huang et al. proved that when the loading angle is 20°, the Brazilian disk specimen will crack in the center, and a central tensile failure will occur, which meets the theoretical requirements for the indirect tensile test [23]. In addition, You, Wang, Huang deduced the calculation formula of the tensile strength measured by a platform Brazilian splitting test and obtained the corresponding calculation formula [23,24,25].

The above researches are of guiding significance for deriving the numerical solution of the indirect tensile strength of the asphalt pavement interlayer bonding material (hereinafter referred to as the indirect tensile strength of the interlayer bond). However, those formulas cannot be directly applied in the calculation of the tensile strength in this work. The main reasons are as follows: firstly, the existing researches are mainly focused on the analysis of the Brazilian disk with the specific platform. The calculation formulas of the tensile strength are not universal. Secondly, most of the researches depend on the finite element or the discrete element simulation for the simulated calculation, lacking a corresponding theoretical analysis. If the stress solutions obtained from these formulas are used to represent the stress state of the samples in this study, deviations from the actual state may occur.

Based on the above analysis, based on the two-dimensional elastic theory and the Flemish solution, a two-dimensional model of indirect tensile test is established with the aid of finite element method to analyze the stress of the bonding interface, so as to obtain a mechanical model of the binding layer indirect tensile test. Besides, the actual stress solution of the sample in the mechanical model is analyzed theoretically, and the calculation formula of the indirect tensile strength of the binding layer is deduced on the premise of a central cracking along with the bonding interface.

## 2. Establishment of the Mechanical Model for the Interlayer Bond Indirect Tensile Test

**Figure 1:**
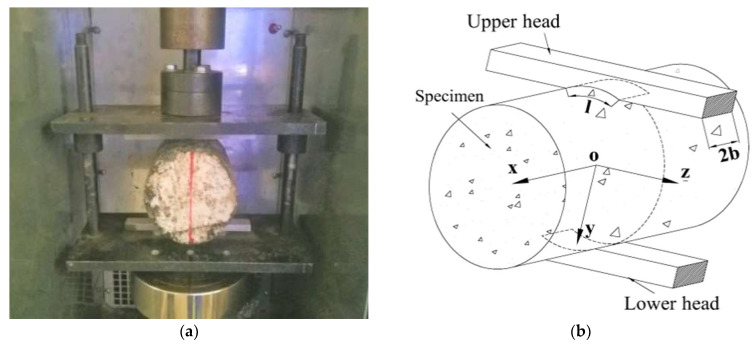
Test: (**a**) loading test; (**b**) loading model.

The specimen and the loading mode used in the test are shown in Figure 1a. The specimen is a cylinder with a diameter of 100 mm, and a height of 100 mm, which is drilled from a double-layer rutting plate specimen. The upper and lower loading strips were parallel positioned on, and under the bonding interface. Since the elastic properties of the indenter and the specimen are different, the friction will generate along with the interface of the two kinds of material (Hooper, 1970) [26]. In order to reduce the additional shear stress caused by the friction, the lubricant was applied on the surfaces of the strips [27]. A radial compression loading was exerted by a universal testing machine. The displacement control mode was adopted with a loading rate of 20 mm/min. Figure 1b shows the loading model diagram, where, the YOZ plane is the bonding interface, and the XOY plane is the surface that passes through the cylinder centroid and is perpendicular to the YOZ plane.

To ensure the rationality and the operability of the test, firstly, the stress distribution on the loading surface must be uniform. Secondly, the internal stress transfer within the specimen is uniform, and the specimen cracks initially on the interlayer bonding surface (i.e., the central plane of the load). Therefore, the following assumptions were proposed:

(1) To optimize the uniform stress distribution, an arc loading is applied on the plane along the *x*-axis. In accordance with Saint-Venant’s principle and the researches done by Kourkoulis et al., this loading mode can effectively abate the stress concentration [28,29,30]. Kourkoulis et al. conducted a series of standard Brazilian disk tests employing a new three-dimensional digital image correlation system. It was found that when the center angle of the arc loading surface was 24°, the stress was evenly distributed along the contact ring [30]. Therefore, the center angle of the arc loading surface in this study is selected 24°.
(2) To ensure the uniform internal stress transfer in the specimen, the specimen should be homogeneously isotropic elastic [31]. When the asphalt mixture specimen is loaded at low temperatures, the material displays elastic properties. Therefore, the specimen can be regarded as homogeneous isotropic [32,33,34].
(3) The bonding layer is the weakest part of the specimen [35,36]. The tensile strength of the other part of the specimen is greater than that of the interlayer bonding surface [37]. Therefore, the sample will start to crack along the YOZ plane.

**Figure 2:**
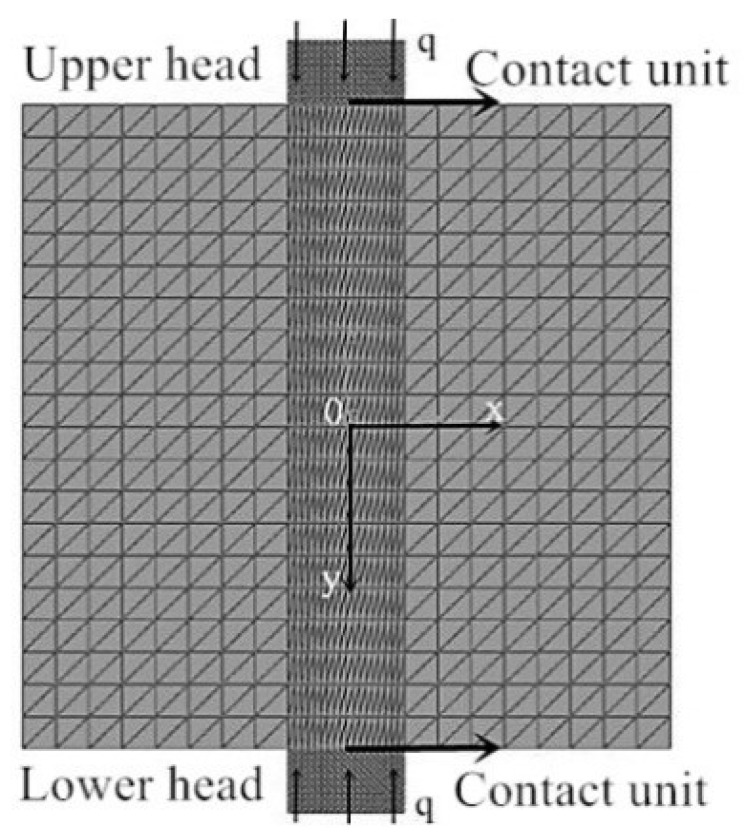
Finite element 2D model of XOY plane of the sample.

**Figure 3:**
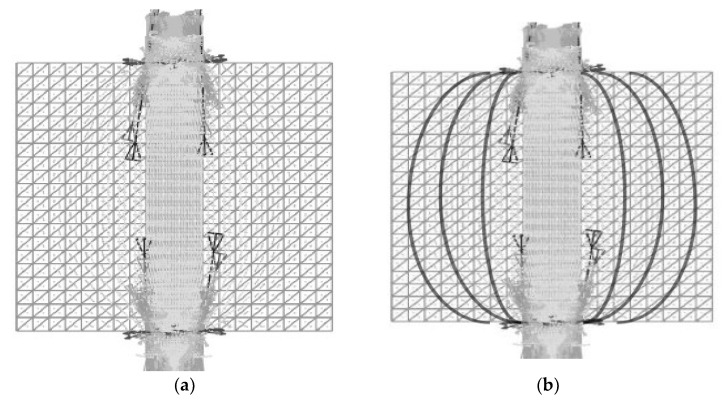
Stress distribution: (**a**) stress distribution results; (**b**) bold display of stress distribution trajectory.

In the following section, a two-dimensional model of the XOY plane, including the loading indenter, is established with the finite element analysis software ABAQUS, as shown in Figure 2. A uniform compression is applied to the specimen, as described in (1). The uniform displacement loading is ensured through the contact units between the indenter and the specimen. The friction coefficient between the contact units is assumed to be 0 due to the application of the lubricants. The elastic modulus E and the Poisson’s ratio are taken as 1.2 GPa and 0.35, respectively, for the rectangular specimen model with a length of 100 mm, and a width of 100 mm. The center angle 2α of the upper and lower indenters is taken as 20°. The elastic moduli of the two indenters are set as 206 Gpa according to the material property. Hence, its elastic deformation can be ignored when compared with the specimen. The Poisson’s ratios of the indenters are set as 0.3. The uniform compressive load *q* is applied on the upper and the lower indenters simultaneously. The value of *q* is calculated using Formula (1), where the test load *P* equals 12 kN [38]. The calculated stress distribution is shown in Figure 3a. The stress analysis was carried out along the XOY plane. In the finite element simulation, a reasonable design of the mechanical model of the XOY plane can ensure that the crack starts from the central point of the plane, which are the key to ensure that the generation and propagation of the cracking is along the YOZ plane.

(1) q=P/2b

where, $2b=R_{0}\tan\alpha$; $l\approx 2R_{0}\sin24$; α is a half of the platform center angle; $R_{0}$ is the radius of the cylindrical specimen, 50 mm.

**Figure 4:**
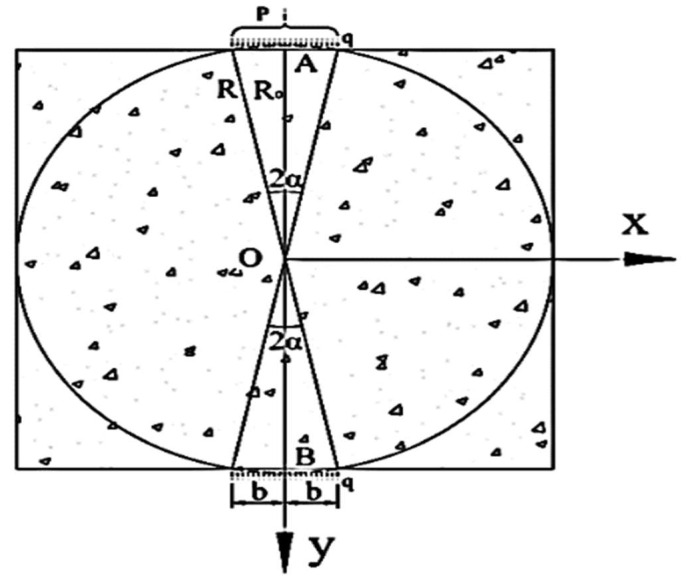
Mechanical model of the indirect tensile test of the interlayer bond.

For the convenience of this discussion, the tensile stress distribution trajectory in Figure 3a is thickened, as shown in Figure 3b. As known from Figure 3b, the maximum horizontal tensile stress $\sigma_{x}$ appears near the XOY plane center of the test piece, and gradually diminishes from the center to the vicinity of the two loading indenters. Since the tensile stresses in the four rectangular corners on the XOY plane are rather small, the tensile stresses in these regions are ignored when the calculation model for the indirect tensile strength of the interlayer bond is established. The mechanical model of the indirect tensile test of the interlayer bond is shown in Figure 4.

On account of the established model, the calculation formula of the indirect tensile strength of the interlayer bond is derived, and the validity of the formula is verified through the experiment in the following section.

## 3. Theoretical Analysis of the Indirect Tensile Test of the Interlayer Bond

In the previous section, the indirect tensile test mechanism of the interlayer bond is discussed. The two-dimensional finite element calculation model is established, and the mechanical model in Figure 4 is proposed. It is assumed that the specimen is a semi-infinite plane body that is subjected to a uniform load on its boundary in seeking the stress solution. The internal stress at any point within the specimen is the superposition of the radial stresses transferred from both the upper and the lower loading heads [39]. The solution of the stress in the specimen is obtained in the light of the elastic mechanics under the premise that the specimen is a semi-infinite plane body. However, the actual specimen is not a semi-infinite plane body. Therefore, it is necessary to obtain a free boundary in the mechanical model. To achieve it, the stress solution obtained at the boundary of the model should be superimposed with a counter stress solution within the specimen. In this way, the theoretical solution of the actual stress within the specimen can be obtained.

### 3.1. Stress Solution on the Boundary of the Indirect Tensile Test Model of the Interlayer Bond

**Figure 5:**
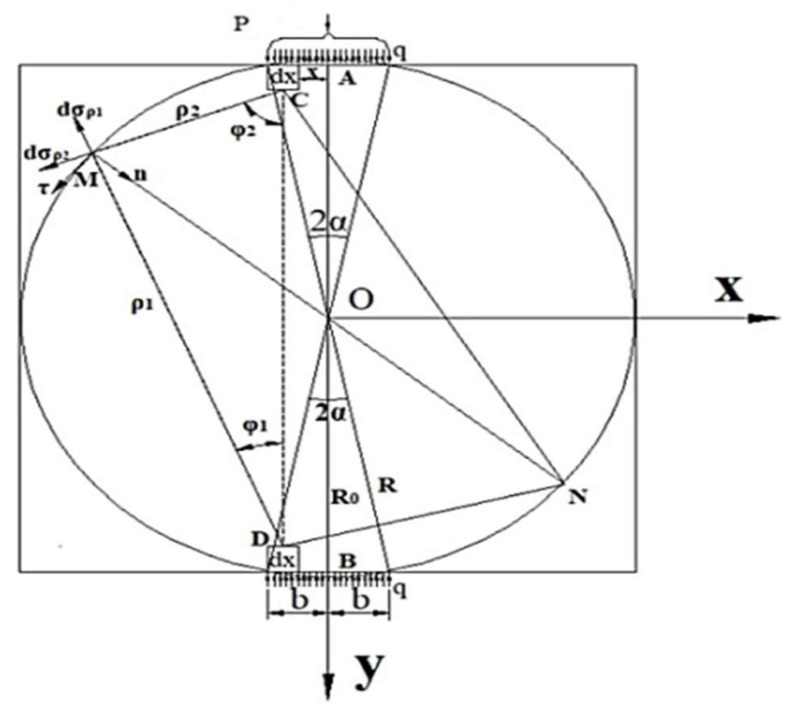
Calculation schematic diagram of the boundary stress mechanical model of the indirect tensile test of the interlayer bond.

To obtain the stress of any point M at the boundary of the indirect tensile test model, a pair of symmetrical elements dx at the upper and the lower loading indenters are selected and named as C and D, whose micro element force dF=qdx is shown in Figure 5. According to the Saint-Venant’s principle, the stress components produced by the two symmetrical microelement forces at the point M in polar coordinates are obtained from the Flamant solution of the semi-infinite plane body which is subjected to the vertical load.

(2) $$d\sigma_{\rho 1}=-\frac{2dF\cos\varphi_{1}}{\pi \rho_{1}},d\sigma_{\rho 2}=-\frac{2dF\cos\varphi_{2}}{\pi \rho_{2}},d\varphi_{1}=d\varphi_{2}=0$$

where, $\rho_{1}$ and $\rho_{2}$ are the radial distance between the microelement and the boundary point M, respectively, $\varphi_{1}$ and $\varphi_{2}$ are the circumferential angles between $\rho_{1}$, $\rho_{2}$ and the vertical direction, respectively.

Through the point M, the diameter MN of the circle O is drawn, which is intersected with the circle O at points M and N. Since the microelement is small enough and the loading angles of the indenters are relatively small, it can be approximated that C and D are both on the circumference of the circle O. According to the circumferential angle theorem, the circumferential angle corresponding to the diameter is a right angle, therefore, ∠MCN=∠MDN≈π/2. In line with the circumferential angle theorem and the central angle theorem, $∠MNC=∠MDC=\varphi_{1}$ and $∠MND=∠MCD=\varphi_{2}$. Then, $∠CMN=\pi /2-\varphi_{1}$ and $∠DMN=\pi /2-\varphi_{2}$. In accordance with the superposition of the coordinate transformation formulas of the stress component in the elastic mechanics, the tangent stress dτ and the normal stress $d\sigma_{n}$ at point M can be obtained as follows:

(3) $$d\tau =(d\sigma_{\rho 1}-d\sigma_{\varphi 1})\sin(\frac{\pi }{2}-\varphi_{2})\cos(\frac{\pi }{2}-\varphi_{2})+(d\sigma_{\rho 2}-d\sigma_{\varphi 2})\sin[-(\pi /2-\varphi_{1})]\;\cdot \cos[-(\pi /2-\varphi_{1})]=-\frac{2dF}{\pi }(\frac{\cos\varphi_{1}}{\rho_{1}}\sin\varphi_{2}\cos\varphi_{2}-\frac{\cos\varphi_{2}}{\rho_{2}}\sin\varphi_{1}\cos\varphi_{1})$$

(4) $$d\sigma_{n}=d\sigma_{\rho 1}\cos^{2}(\pi /2-\varphi_{2})+d\sigma_{\rho 2}\cos^{2}[-(\pi /2-\varphi_{1})]\;=(\frac{\cos\varphi_{1}\sin^{2}\varphi_{2}}{\rho_{1}}+\frac{\cos\varphi_{2}\sin^{2}\varphi_{1}}{\rho_{2}})$$

In line with the triangle theorem, Formula (5) exists in Δ*MDN* and Δ*MCN*:

(5) $$\left\{\begin{array}{c} \rho_{1}=2R\cos(\rho /2-\varphi_{2})=2R\sin\varphi_{2}\\ \rho_{2}=2R\cos(\rho /2-\varphi_{1})=2R\sin\varphi_{1} \end{array}\right.$$

By introducing Formula (5) into Formulas (3) and (4), Formula (6) can be deduced as:

(6) $$d\sigma_{n}=-\frac{dF}{\pi R}\sin(\varphi_{1}+\varphi_{2}),\;d\tau =0$$

In Δ*MCD*, $∠CMD=\pi -(\varphi_{1}+\varphi_{2})$, according to the sine theorem of the triangle, the following relationship exist:

(7) $$\rho_{2}/\sin\varphi_{1}=\frac{2R_{0}}{\sin[\pi -(\varphi_{1}+\varphi_{2})]}$$

In Δ*MCN*, $\rho_{2}/\sin\varphi_{1}=2R$, and $R=R_{0}/\cos\alpha$, which are substituted into Formula (7) respectively, and Formula (8) are obtained:

(8) $$\sin[\pi -(\varphi_{1}+\varphi_{2})]=\sin(\varphi_{1}+\varphi_{2})=\cos\alpha$$

If Formula (8) is substituted into Formula (6), the following formula is obtained:

(9) $$d\sigma_{n}=-\frac{dF}{\pi R}\cos\alpha =-\frac{dF}{\pi R_{0}}\cos^{2}\alpha$$

Therefore, the stress at any point on the boundary of the indirect tensile test model of the bonding layer can be established by integrating $d\sigma_{n}$ along the loading head region under the uniform load *q*:

(10) $$\sigma_{n}=\int \begin{array}{c} R\sin\alpha \\ -R\sin\alpha \end{array}d\sigma_{n}=\int \begin{array}{c} R\sin\alpha \\ -R\sin\alpha \end{array}\frac{P}{2bl}\frac{-\cos^{2}\alpha }{\pi R_{0}}dx=-\frac{P\cos^{2}\alpha }{\pi R_{0}l}$$

As is shown by Equation (10), there exists one constant pressure stress $P\cos^{2}\alpha /\pi R_{0}l$ along the boundary of the indirect tensile test model of the interlayer bond, where, the tensile stress is positive and the compressive stress is negative. To maintain the assumed stress distribution in the model, a uniform compressive stress field with the intensity of $P\cos^{2}\alpha /\pi R_{0}l$ needs to be applied on the model boundary. In addition, the theoretical derivation in this paper is based on the elastic semi-infinite plane body, while the specimen in the actual test has a free boundary. Therefore, to ensure the rationality of the calculation, it is necessary to superimpose a uniform stress field with the intensity of $P\cos^{2}\alpha /\pi R_{0}l$ on the stress solution of the theoretical model so as to match the actual free boundary conditions.

### 3.2. Stress Solution in the Indirect Tensile Test Model of the Interlayer Bond

**Figure 6:**
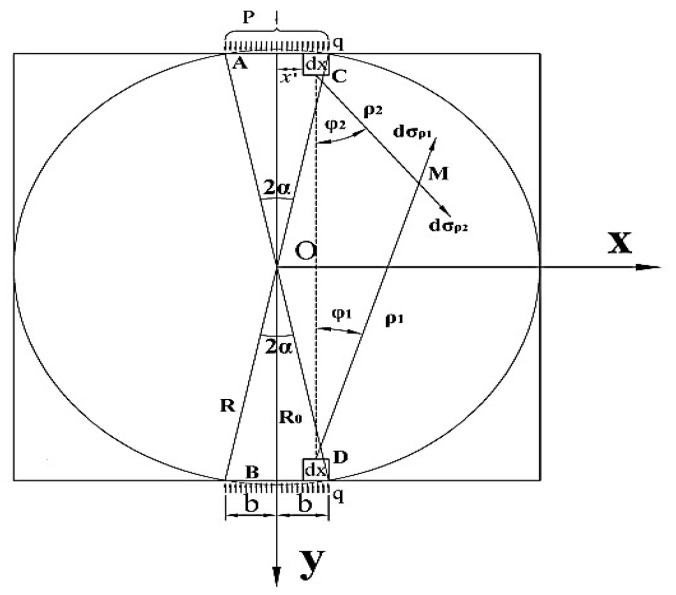
Schematic diagram of the internal stress calculation in the mechanical model of the indirect tensile test of the interlayer bond.

Any point M in the indirect tensile test model of the interlayer bond is shown in Figure 6. Its stress component in polar coordinates can be expressed by Formula (2). By means of a coordinate transformation and the stress superposition in the same direction, the stress component of any microelement in the model in a rectangular coordinate system can be obtained:

(11) $$\{\begin{array}{c} d\sigma_{x}=d\sigma_{\rho 1}\cos^{2}(\pi /2-\varphi_{1})+d\sigma_{\rho 2}\cos^{2}[-(\pi /2-\varphi_{2})]=-\frac{2dF}{\pi }\\ \cdot (\frac{\cos\varphi_{1}\sin^{2}\varphi_{1}}{\rho_{1}}+\frac{\cos\varphi_{2}\sin^{2}\varphi_{2}}{\rho_{2}})\\ d\sigma_{y}=d\sigma_{\rho 1}\sin^{2}(\pi /2-\varphi_{1})+d\sigma_{\rho 2}\sin^{2}[-(\pi /2-\varphi_{2})]=-\frac{2dF}{\pi }\\ \cdot (\frac{\cos^{3}\varphi_{1}}{\rho_{1}}+\frac{\cos^{3}\varphi_{2}}{\rho_{2}})\\ d\tau_{xy}=d\sigma_{\rho 1}\sin(\pi /2-\varphi_{1})\cos(\pi /2-\varphi_{1})+d\sigma_{\rho 2}\sin[-(\pi /2-\varphi_{2})]\\ \cdot \cos[-(\pi /2-\varphi_{2})]=-\frac{2dF}{\pi }(\frac{\cos^{2}\varphi_{1}\sin\varphi_{1}}{\rho_{1}}-\frac{\cos^{2}\varphi_{2}\sin\varphi_{2}}{\rho_{2}}) \end{array}$$

In the model, there exists a trigonometric relationship, which can be expressed as follows:

(12) $$\{\begin{array}{c} \sin\varphi_{1}=(x-x^{\prime })/\rho_{1},\cos\varphi_{1}=(R_{0}-y)/\rho_{1}\\ \rho_{1}{}^{2}={\left(x-x^{\prime }\right)}^{2}+{\left(R_{0}-y\right)}^{2}\\ \sin\varphi_{2}=(x-x^{\prime })/\rho_{2},\cos\varphi_{2}=(R_{0}+y)/\rho_{2}\\ \rho_{2}{}^{2}={\left(x-x^{\prime }\right)}^{2}+{\left(R_{0}+y\right)}^{2} \end{array}$$

By substituting Formula (12) is substituted into Formula (11). The results obtained are integrated along the loading indenter region under the uniform load. When the uniform tensile stress $P\cos^{2}\alpha /\pi R_{0}l$ is superimposed on the boundary, the stress components in the mechanical model of the indirect tensile test can be obtained as follows:

(13) $$\begin{array}{c} \sigma_{x}=-\frac{P\cos\alpha }{\pi R_{0}l}\int \begin{array}{c} R\sin\alpha \\ -R\sin\alpha \end{array}\left\{\frac{(R_{0}-y){\left(x-x^{\prime }\right)}^{2}}{{\left[{\left(x-x^{\prime }\right)}^{2}+{\left(R_{0}-y\right)}^{2}\right]}^{2}}+\frac{(R_{0}+y){\left(x-x^{\prime }\right)}^{2}}{{\left[{\left(x-x^{\prime }\right)}^{2}+{\left(R_{0}+y\right)}^{2}\right]}^{2}}\right\}\\ +\frac{P\cos^{2}\alpha }{\pi R_{0}l}=\frac{P\cos^{2}\alpha }{2\pi R_{0}l}(\frac{B_{1}}{A_{1}}+C_{1}+\frac{B_{2}}{A_{2}}-C_{2}+\frac{B_{3}}{A_{3}}+C_{3}+\frac{B_{4}}{A_{4}}-C_{4})+\frac{P\cos^{2}\alpha }{\pi R_{0}l} \end{array}$$

(14) $$\begin{array}{c} \sigma_{y}=-\frac{P\cos\alpha }{\pi R_{0}l}\int \begin{array}{c} R\sin\alpha \\ -R\sin\alpha \end{array}\left\{\frac{{\left(R_{0}-y\right)}^{3}}{{\left[{\left(x-x^{\prime }\right)}^{2}+{\left(R_{0}-y\right)}^{2}\right]}^{2}}+\frac{{\left(R_{0}+y\right)}^{3}}{{\left[{\left(x-x^{\prime }\right)}^{2}+{\left(R_{0}+y\right)}^{2}\right]}^{2}}\right\}\\ +\frac{P\cos^{2}\alpha }{\pi R_{0}l}=-\frac{P\cos^{2}\alpha }{2\pi R_{0}l}(\frac{B_{1}}{A_{1}}-C_{1}+\frac{B_{2}}{A_{2}}+C_{2}+\frac{B_{3}}{A_{3}}-C_{3}+\frac{B_{4}}{A_{4}}+C_{4})+\frac{P\cos^{2}\alpha }{\pi R_{0}l} \end{array}$$

(15) $$\begin{array}{c} \tau_{\mathrm{xy}}=-\frac{P\cos\alpha }{\pi R_{0}l}\int \begin{array}{c} R\sin\alpha \\ -R\sin\alpha \end{array}\left\{\frac{{\left(R_{0}-y\right)}^{2}(x-x^{\prime })}{{\left[{\left(x-x^{\prime }\right)}^{2}+{\left(R_{0}-y\right)}^{2}\right]}^{2}}-\frac{{\left(R_{0}+y\right)}^{2}(x-x^{\prime })}{{\left[{\left(x-x^{\prime }\right)}^{2}+{\left(R_{0}+y\right)}^{2}\right]}^{2}}\right\}\\ \cdot dx^{\prime }+\frac{P\cos^{2}\alpha }{\pi R_{0}l}=-\frac{P\cos^{2}\alpha }{2\pi R_{0}l}[\frac{{\left(R_{0}+y\right)}^{2}}{A_{1}}-\frac{{\left(R_{0}+y\right)}^{2}}{A_{2}}-\frac{{\left(R_{0}-y\right)}^{2}}{A_{3}}+\frac{{\left(R_{0}-y\right)}^{2}}{A_{4}}] \end{array}$$

where,

(16) $$\{\begin{array}{c} A_{1}={\left(R_{0}+y\right)}^{2}+{\left(x-R_{0}\tan\alpha \right)}^{2}\\ A_{2}={\left(R_{0}+y\right)}^{2}+{\left(x+R_{0}\tan\alpha \right)}^{2}\\ A_{3}={\left(R_{0}-y\right)}^{2}+{\left(x-R_{0}\tan\alpha \right)}^{2}\\ A_{4}={\left(R_{0}-y\right)}^{2}+{\left(x+R_{0}\tan\alpha \right)}^{2}\\ B_{1}=(R_{0}+y)(R_{0}\tan\alpha -x),B_{2}=(R_{0}+y)(R_{0}\tan\alpha +x)\\ C_{1}=\arctan(\frac{x-R_{0}\tan\alpha }{R_{0}+y}),C_{2}=\arctan(\frac{x+R_{0}\tan\alpha }{R_{0}+y})\\ B_{3}=(R_{0}-y)(R_{0}\tan\alpha -x),B_{4}=(R_{0}-y)(R_{0}\tan\alpha +x)\\ C_{3}=\arctan(\frac{x-R_{0}\tan\alpha }{R_{0}-y}),C_{4}=\arctan(\frac{x+R_{0}\tan\alpha }{R_{0}-y}) \end{array}$$

### 3.3. Tensile Strength of the Interlayer Bond in the Indirect Tensile Test Model

**Figure 7:**
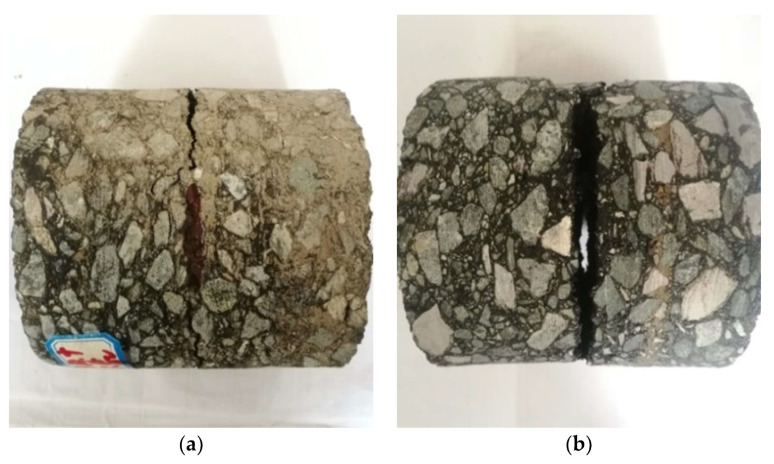
Interlayer bond failure of the indirect tensile test specimen: (**a**) SBR modified emulsified asphalt sample; (**b**) SW binder sample.

Figure 7 shows the interlayer bond failure of the indirect tensile specimens. In line with the above experiment design, the specimens were destroyed from the bond layer in the form of the center cracking. The secondary oblique shear cracks near the loading indenter were caused by the friction between the indenter and the specimen due to the different elastic properties of the two materials [40]. To avoid or mitigate the damage caused by secondary cracking, the lubricant was applied to the surfaces between the indenter and the specimen. The experimental results show that the experiment’s design is characterized by feasibility.

On the loading diameter of the specimen, where x=0, the shear stress *τ_xy_* is 0, as is calculated from Formula (15). Therefore, it is known that the horizontal stress $\sigma_{x}$ and the vertical stress $\sigma_{y}$ on the loading diameter are the maximum and the minimum principal stresses, respectively, which can be calculated from Formulas (13) and (14):

(17) $$\{\begin{array}{c} \sigma_{1}=\sigma_{x}=\frac{P\cos^{2}\alpha }{\pi R_{0}l}(\frac{B_{1}}{A_{1}}+C_{1}+\frac{B_{3}}{A_{3}}+C_{3})+\frac{P\cos^{2}\alpha }{\pi R_{0}l}\\ \sigma_{3}=\sigma_{y}=\frac{-P\cos^{2}\alpha }{\pi R_{0}l}(\frac{B_{1}}{A_{1}}-C_{1}+\frac{B_{3}}{A_{3}}-C_{3})+\frac{P\cos^{2}\alpha }{\pi R_{0}l} \end{array}$$

where,

(18) $$\{\begin{array}{c} A_{1}=A_{2}={\left(R_{0}+y\right)}^{2}+{\left(R_{0}\tan\alpha \right)}^{2}\\ A_{3}=A_{4}={\left(R_{0}-y\right)}^{2}+{\left(R_{0}\tan\alpha \right)}^{2}\\ B_{1}=B_{2}=(R_{0}+y)R_{0}\tan\alpha ,B_{3}=B_{4}=(R_{0}-y)R_{0}\tan\alpha \\ C_{1}=-C_{2}=-\arctan[(R_{0}\tan\alpha )/(R_{0}+y)]\\ C_{3}=-C_{4}=-\arctan[(R_{0}\tan\alpha )/(R_{0}-y)] \end{array}$$

In fact, the interlayer bond failure in the asphalt pavement is typically caused by the maximum shear stress. According to the Tresca yield criterion, no matter what the stress state is, as long as the maximum shear stress $\tau_{\max}$ reaches the limit shear stress $\tau_{0}$ under unidirectional stress state (its magnitude is only related to the material properties), the yield failure will occur [41]. When $\sigma_{1}\geq \sigma_{2}\geq \sigma_{3}$, the formula can be expressed as:

(19) $$\tau_{\max}=\frac{\sigma_{S}}{2}$$

where, $\sigma_{S}$ is the Tresca ultimate stress of the cross-section normal stress. According to the strength criterion of the Tresca yield law, the tensile strength of the indirect tensile test for the interlayer bond should satisfy Formula (20):

(20) $$\sigma_{1}-\sigma_{3}=\sigma_{S}\geq \sigma_{T}$$

**Figure 8:**
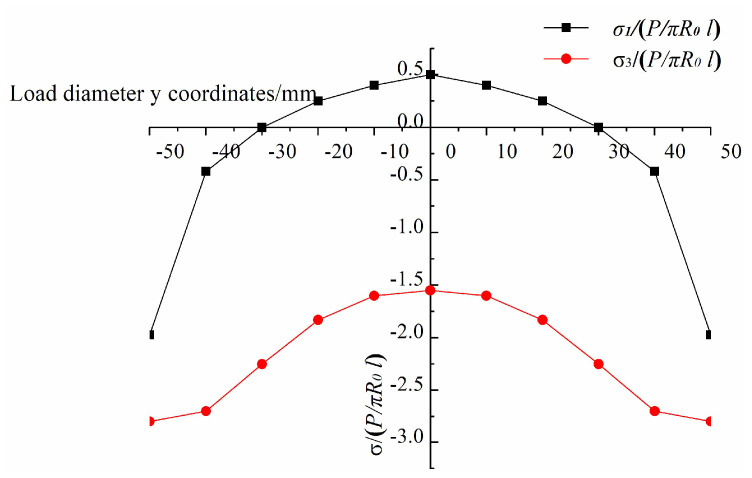
Dimensionless $\sigma_{1}$ and $\sigma_{3}$ along the loaded diameters.

To obtain the critical condition for the Tresca yield criterion, the finite element calculation results are extracted, and the dimensionless values $\left(\sigma /(P/\pi R_{0}l)\right)$ of $\sigma_{1}$ and $\sigma_{3}$ of the line AB on the XOY plane are plotted in Figure 8. As is known from Figure 8, $\sigma_{1}\geq \sigma_{2}\geq \sigma_{3}$ is always true in the experiment designed in this paper, so Formula (17) is substituted into Formula (20) to obtain:

(21) $$\sigma_{1}-\sigma_{3}=\frac{2P\cos^{2}\alpha }{\pi R_{0}l}(\frac{B_{1}}{A_{1}}+\frac{B_{3}}{A_{3}})\geq \sigma_{T}$$

When the stress reaches the Tresca limit stress, i.e., $\sigma_{S}=\sigma_{T}$, the specimen is considered to have been damaged [42]. According to the Tresca yield criterion and the test results, the specimen begins to fail from the center of the XOY plane. Since x=0 and y=0 at the coordinate origin, as is known from Formula (18), $A_{1}=A_{3}=R_{0}{}^{2}(1+\tan\alpha )$ and $B_{1}{=B}_{3}=R_{0}{}^{2}\tan\alpha$. According to Formula (21), the calculation formula for the tensile strength of the interlayer bond in the indirect tensile test is as follows:

(22) $$\sigma_{T}=\frac{4P\cos^{2}\alpha \tan\alpha }{\pi R_{0}l(1+\tan\alpha )}$$

And since $l\approx 2R_{0}\sin24$ and α=20°, the final calculation formula for the tensile strength of the interlayer bond in the indirect tensile test is obtained:

(23) $$\sigma_{T}=\frac{0.3688P}{R_{0}{}^{2}}$$

## 4. Experimental Verification of the Indirect Tensile Test of the Interlayer Bond

### 4.1. Design of the Test

**Figure 9:**
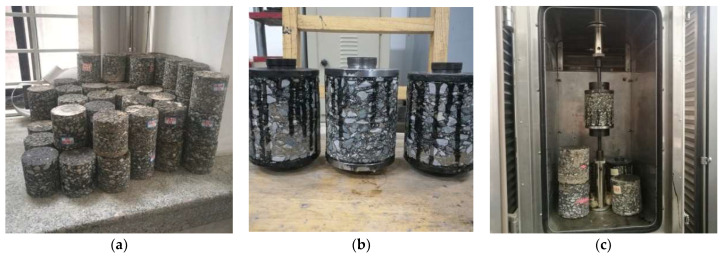
Specimens of bonding materials used for test analysis: (**a**) shaped specimens; (**b**) installation of tensile specimens; (**c**) specimens kept in the environmental chamber.

To verify the rationality and the operability of the interlayer bond indirect tensile test designated in this work, the following experiment was designed and conducted. Three kinds of bonding materials, the Styrene-Butadiene Rubber (SBR) modified emulsified asphalt, the Styrenic Block Copolymers (SBS) modified emulsified asphalt, and the SBR + waterborne epoxy resin (SW) were utilized. Sixteen double-layered specimens were cored from the rutting plates manufactured for each kind of bonding material, with four specimens being as one group, as shown in Figure 9a. The direct tensile test was set as the control group. In the direct tensile test, the drawing head was bonded to the upper and the lower surfaces of the specimen with strong adhesive resin, as shown in Figure 9b. After the resin was cured, the direct tensile test was carried out with a loading rate of 20 mm/min. The other two groups of specimens were applied for the indirect tensile test with the loading rate of 20 mm/min as well. These specimens were kept at the specified temperature, 5 °C and 10 °C respectively, for more than 5 h, and were then loaded at the specified temperature, as shown in Figure 9c.

### 4.2. Analysis of Test Results

**Figure 10:**
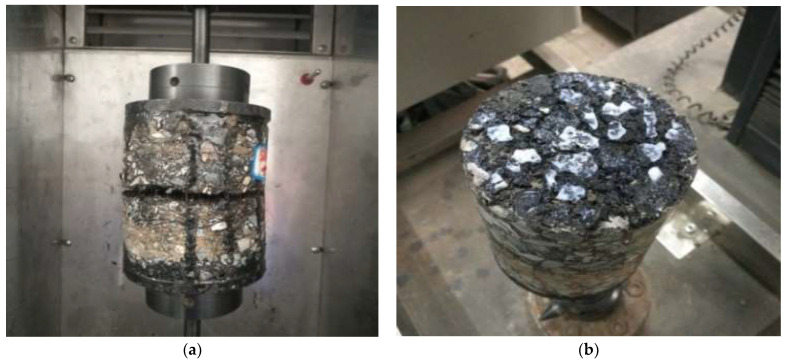
Direct tensile test of the interlayer bond: (**a**) the direct tensile failure; (**b**) the detachment between the drawing head and the specimen.

The typical direct tensile failure of the specimens is shown in Figure 10a. The specimens cracked at the interlayer bonding surface under normal circumstances. During the test procedure, some specimens detached from the interface between the drawing head and the specimens and had to be attached again, as shown in Figure 10b. The indirect tensile failure mode of the specimen is shown in Figure 7.

The mean values of the indirect tensile strength and the direct tensile strength of the three bonding materials at the two temperatures are shown in Table 1.

The deviation factor is analyzed for the data in Table 1 using Formula (24), and the analysis results are shown in Table 2.

(24) $$C_{V}=\frac{\sigma }{\left|\mu \right|}\times 100\%$$

where, σ is the standard deviation of the test data and |μ| is the absolute value of the sample data average.

Table 1 is selected to be 5 °C and 10 °C rather than higher temperatures. The temperatures are chosen in accordance with the following considerations. First, the tensile strength of the interlayer bond will decrease sharply with large discreteness because the material is no longer elastic at higher temperatures. Apart from that, when the temperature rises, the asphalt mixture on and below the interlayer bond loosens with a consequent degradation in the interface adhesion and the friction [43,44]. Its mechanical performance will fluctuate or redistribute, even go beyond the elastic limit [45]. Therefore, higher temperatures are not selected in this test.

As shown in Table 1, the indirect tensile strength of the three types of bonding materials at 5 °C and 10 °C is greater than their corresponding direct tensile strength. The indirect tensile strength average is about 1.48 times that of the direct tensile strength at 5 °C, and, 1.51 times at 10 °C. The above differential is mainly related to the stress transmission and redistribution in the specimens during the loading period [43]. This effect is more significant for the direct tensile loading due to the longer stress transfer path [46,47].

**Figure 11:**
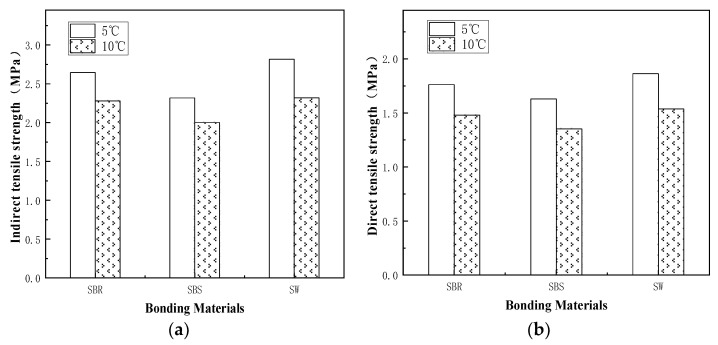
Comparison of tensile strength of bonding materials: (**a**) indirect tensile test; (**b**) direct tensile test.

To explain the relationship between the indirect tensile strength and the direct tensile strength of the three kinds of binder more intuitively, the test data are plotted as a column chart, as shown in Figure 11. It can be seen from Figure 11b that the average value of the direct tensile strength of the three materials is SW > SBR > SBS at 5 °C and 10 °C. Among them, SW has the greatest direct tensile strength due to the optimum material composition. SBR shows better low-temperature tensile properties than SBS, which proves the rationality of the designed indirect tensile test and the correctness of the derived formula. Moreover, the specimen and the drawing head fell off frequently during the direct tensile test, as shown in Figure 10b. This not only prolonged the test cycle and aggregated the workload, but also could lead to a waste of specimens and resources. Therefore, the indirect tensile test can better replace the direct tensile test at low temperatures.

The deviation coefficient can express the degree of the data discreteness after the measurement scale differential between the data is eliminated [48]. As known from Table 2, the average deviation coefficient of the indirect tensile strength is below 9%, while the coefficient of the direct tensile strength is greater than 12%. Among them, the deviation coefficients of the SBR and SBS are greater than 15%, which is beyond the reasonable range required in the data statistical analysis [49]. This indicates that the direct tensile test results have displayed great dispersion, while the dispersion of indirect tensile test results is within a reasonable range.

## 5. Conclusions

In view of the defects of the direct tensile test for the interlayer bond in the asphalt pavement, the indirect tensile strength test was designed and the corresponding calculation formula was deduced. The validity of the test and the formula were verified.

I. The interlayer bond strength of asphalt pavement can be determined by an indirect tensile test, which effectively reduces the workload of the direct tensile test and the waste of resources.
II. The finite element or discrete element theoretical solution was utilized in most existing researches to express the stress state in the specimen. Different from these researches, based on the two-dimensional elastic theory, the calculation formula of indirect tensile strength of the interlayer bond is derived in this paper. The calculation formula supplements the deficiency of the mechanical theoretical analysis of the indirect tensile test of the interlaminar bind.
III. The failure mode of the specimen verifies the validity of the test introduce, i.e., under the indirect tensile loading conditions designed, the crack starts from the center of the XOY plane in the specimen.
IV. At 5 °C and 10 °C, the ranking of the direct tensile strength for the three bonding is SW > SBR > SBS. This sequence is also confirmed in the indirect tensile test, which reduces the workload and the waste of materials. Therefore, the indirect tensile test can better replace the direct tensile test at low temperatures. It also conforms to the premise of the theoretical deduction.
V. The indirect tensile test at low temperatures can better diminish the deviation of the test data. The main reason is that the temperature change can caus a stress redistribution in the sample. Due to the existence of internal pores of asphalt mixture, the stress redistribution could affect the internal stress transmission in the sample. Since the stress transmission path under the direct tensile mode is longer, therefore, the impact is more significant than that under indirect tensile mode. That’s why the deviation coefficient of the direct tensile test data is greater than that of the indirect tensile test.

## Figures and Tables

**Table 1:** Interlayer bond tensile strength of the indirect tensile test and the direct tensile test.

Temperatures	Bonding Materials	Indirect Tensile Strength (MPa)	Direct Tensile Strength (MPa)
5 °C	SBR	2.645	1.762
	SBS	2.318	1.629
	SW	2.816	1.863
10 °C	SBR	2.279	1.480
	SBS	2.001	1.352
	SW	2.319	1.537

**Table 2:** Strength deviation coefficient of the two experimental approaches.

Items	Temperatures	Deviation Coefficient $C_{V}$ (%)			
		SBR	SBS	SW	Mean Value
Indirect tensile strength	5 °C	4.96	4.13	3.02	4.04
	10 °C	9.92	8.88	7.47	8.76
Direct tensile strength	5 °C	15.49	16.77	12.80	15.02
	10 °C	16.20	17.14	12.21	15.18

## Data Availability

The data presented in this study are available on request form the corresponding author.

## References

[1] Li X.-L., Liu X.-Y., Lv X.-C., Ye J.-H. (2019). Experimental on indirect tensile test for asphalt mixture based on discrete element simulation and fractal dimension. J. Chang. Univ. Nat. Sci. Ed..

[2] Mu K., Gao Z., Shi X., Li Y. (2020). Interface Behavior of Asphalt Pavements Constructed by Conventional and Double-Decked Paving Methods. Materials.

[3] Zhang Q., Xu Y.-H., Wen Z.-G. (2017). Influence of water-borne epoxy resin content on performance of waterborne epoxy resin compound SBR modified emulsified asphalt for tack coat. Constr. Build. Mater..

[4] Zhang R., Guo L., Li W.-J. (2021). Combining Thermal Loading System with Acoustic Emission Technology to Acquire the Complete Stress-Deformation Response of Plain Concrete in Direct Tension. Materials.

[5] Liao W.-C., Chen P.-S., Hung C.-W., Wagh S.K. (2020). An Innovative Test Method for Tensile Strength of Concrete by Applying the Strut-and-Tie Methodology. Materials.

[6] Roozbahany E.G., Partl M.N., Witkiewicz P.J. (2013). Fracture testing for the evaluation of asphalt pavement joints. Road Mater. Pavement Des..

[7] Hondros G. (1959). The evaluation of Poisson’s ratio and the modulus of materials of low tensile resistance by the Brazilian (indirect tensile) test with particular reference to concrete. Aust. J. Appl. Sci..

[8] Teng S.-Y., Yang S.-Q., Huang Y.-H., Tian W.-L. (2018). Experimental study of influence of crack filling on mechanical properties of Brazilian disca. Rock Soil Mech..

[9] Dębska B., Lichołai L., Silva G.J.B., Altoé Caetano M. (2020). Assessment of the Mechanical Parameters of Resin Composites with the Addition of Various Types of Fibres. Materials.

[10] Ren J., Dang F., Wang H., Xue Y., Fang J. (2018). Enhancement mechanism of the dynamic strength of concrete based on the energy principle. Materials.

[11] Guo H., Aziz N., Schmidt L. (1993). Rock fracture-toughness determination by the Brazilian test. Eng. Geol..

[12] Zhao T.-B., Zhang W., Xing M.-L., Qiu Y., Yao J.-P. (2021). Study on Determination Method of Rock Tensile Modulus of Brazilian Disc Splitting Test Based on Digital Speckle Correlation Method (DSCM). Arab. J. Sci. Eng..

[13] Zhang G.-D. (1981). Brittle Fracture of Rock Under Dynamic Load. Chin. J. Geothchnical Eng..

[14] Wang Q.Z., Li W., Xie H.P. (2009). Dynamic split tensile test of Flattened Brazilian Disc of rock with SHPB setup. Mech. Mater..

[15] Wang S.-Y. (2019). ; Test Method for Tensile Strength and Fracture Toughness of Sandstone.

[16] Lin H., Xiong W., Yan Q.-X. (2016). Modified Formula for the Tensile Strength as Obtained by the Flattened Brazilian Disk Test. Rock Mech. Rock Eng..

[17] Guo X., Wang X.-B., Bai X.-Y., Wang C.-W., Qi D.-L. (2017). Numerical simulation of effects of loading types and tensile strengths on Brazilian disk test by use of a continuum-discontinuum method. Rock Soil Mech..

[18] Xu Z.-L. (2018). A Concise Course in Elasticity.

[19] QI Y.-Z., ZHANG H., Ji-Xin G. (2018). Experimental Study and Numerical Simulation of Split Tensile Properties and Crack Propagation of Concrete. Three Gorges Univ. Nat. Sci..

[20] Wang M., Cao P. (2015). Numerical study on flattened Brazilian Test and its empirical formula. Electron. J. Geotech. Eng..

[21] Wang M., Cao P. (2016). Numerical analysis of flattened Brazilian disc test based on the cusp catastrophe theory. Math. Probl. Eng..

[22] Khavari P., Heidari M. (2016). Numerical and experimental studies on the effect of loading angle on the validity of flattened Brazilian disc test. J. Geol. Min. Res..

[23] Huang Y.-G., Wang L.-G., Chen J.-R., Zhang J.-H. (2015). Theoretical analysis of flattened Brazilian splitting test for determining tensile strength of rock. Chinses J. Rock Mech. Eng..

[24] You M.-Q., Su C.-D. (2004). Split test of flattend rock disk and related theory. Rock Soil Mech..

[25] Wang Q.-Z., Jia X.-M. (2002). Determination of elastic modulus, tensile strength and fracture toughness of brittle rocks by using flttened brazilian disk specimen—Part 1: Analytical and numerical results. Chinses J. Rock Mech. Eng..

[26] LIAO L., WU X.-T. (2017). Numerical simulatiao of dynamic indirect tensile test for concrete material. J. Hefei Univ. Technol..

[27] Matbuly M.S. (2008). Analysis of mode III crack perpendicular to the interface between two dissimilar strips. Acta Mech. Sin..

[28] Kourkoulis S., Markides C.F., Chatzistergos P. (2012). The Brazilian disc under parabolically varying load: Theoretical and experimental study of the displacement field. Int. J. Solids Struct..

[29] Markides C.F., Kourkoulis S. (2012). The stress field in a standardized Brazilian disc: The influence of the loading type acting on the actual contact length. Rock Mech. Rock Eng..

[30] Kourkoulis S., Markides C.F., Chatzistergos P. (2013). The standardized Brazilian disc test as a contact problem. Int. J. Rock Mech. Min. Sci..

[31] Armitsu Y., Nishioka K., Senda T. (1994). Analysis of Anisotropic Elasticity by Means of Internal Stress in a Reference Isotropic Elastic Body. J. Appl. Math. Mech..

[32] Arshadi A. (2013). Importance of asphalt binder properties on rut resistance of asphalt mixture. Master’s Thesis.

[33] Arand W., Yin Z.-R. (1989). Low temperature characteristics of asphalt mixture. Pet. Asph..

[34] Hu L.-Y. (2019). Effect of anisotropy and viscoelastic properties of asphalt mixtures on the response of asphalt pavements. Master’s Thesis.

[35] Hui B., Zhou B.-W., Wang Z. (2018). The Impact on the Composite Pavement Interlayer Bond Strength of Grooving Parameters. Highw. Eng..

[36] Canestrari F., Ferrotti G., Lu X., Millien A., Partl M.-N., Petit C., Phelipot-Mardelé A., Piber H., Raab C. (2013). Mechanical testing of interlayer bonding in asphalt pavements. Adv. Interlab. Test. Eval. Bitum. Mater..

[37] LIU H.-P., AI C.-F., Rahman A., Gao X.-W., Qiu Y.-J. (2017). Characterization of interlayer bonding in asphalt pavement based on direct tension test with horizontal loading. J. Chang. Univ. Nat. Sci. Ed..

[38] WANG F.-Y., LI Y.-L., REN L.-F. (2009). Study on indirect tensile test parameters of asphalt mixture under dynamic load. J. Transp. Inf. Saf..

[39] Rickmer M., Christian Löbbe A., Erman T. (2019). Stress state analysis of radial stress superposed bending. Int. J. Precis. Eng. Manuf..

[40] Wetscher F., Stock R., Pippan R. (2007). Changes in the mechanical properties of a pearlitic steel due to large shear deformation. Mater. Sci. Eng. A.

[41] Tresca H. (1864). On the flow of solid bodies subjected to high pressures. C. R. l’Acad. Des. Sci..

[42] Mo P.Q., Marshall A.M., Yu H.S. (2014). Elastic-plastic solutions for expanding cavities embedded in two different cohesive-frictional materials. Int. J. Numer. Anal. Methods Geomech..

[43] Maliszewski M., Zofka A., Maliszewska D., Sybilski D., Salski B., Karpisz T., Rembelski R. (2021). Full-Scale Use of Microwave Heating in Construction of Longitudinal Joints and Crack Healing in Asphalt Pavements. Materials.

[44] Shi X., Liu X.-Y., Amir T., Erik S. (2021). Experimental Investigation of the Performance of a Hybrid Self-Healing System in Porous Asphalt under Fatigue Loadings. Materials.

[45] Cheng Y., Wang W., Tan G., Shi C. (2018). Assessing high- and low-temperature properties of asphalt pavements incorporating waste oil shale as an alternative material in jilin province, China. Sustainability.

[46] Shahrul S., Mohammed B.S., Wahab M.M.A., Liew M.S. (2021). Mechanical Properties of Crumb Rubber Mortar Containing Nano-Silica Using Response Surface Methodology. Materials.

[47] Calabrese A.S., D’Antino T., Colombi P., Poggi C. (2021). Low- and High-Cycle Fatigue Behavior of FRCM Composites. Materials.

[48] Xiao Y., Zhang Y., Lu J., Liu Y., Cheng W. (2018). Experimental Analysis on Pre-Stress Friction Loss of Crushed Limestone Sand Concrete Beams. Appl. Sci..

[49] Ograjenek I. (2021). Statistical Analysis of Survey Data.

